# Online public attention toward allergic rhinitis in Wuhan, China: Infodemiology study using Baidu index and meteorological data

**DOI:** 10.3389/fpubh.2022.971525

**Published:** 2022-10-03

**Authors:** Yunfei Wang, Ziang Gao, Hao Lv, Yu Xu

**Affiliations:** Department of Otolaryngology-Head and Neck Surgery, Renmin Hospital of Wuhan University, Wuhan, China

**Keywords:** Baidu index, meteorological data, allergic rhinitis, pollen allergy, Mites, COVID-19

## Abstract

**Background:**

With the popularization of the Internet and medical knowledge, more and more people are learning about allergic rhinitis (AR) on the Internet.

**Objective:**

This study aims to analyze the epidemiological characteristics and online public attention to AR in Wuhan, China, utilizing the most popular search engine in mainland China and meteorological data of Wuhan.

**Methods:**

To study the Internet attention and epidemiological characteristics of AR in Wuhan, the search volume (SV) of “Allergic Rhinitis” in Mandarin and AR-related search terms from 1 January 2014 through 31 December 2021 were recorded. For user interest, the search and demand data were collected and analyzed.

**Results:**

The yearly average Baidu SV of AR in both Wuhan and China increased year by year but began to decline gradually after the COVID-19 pandemic. Baidu SV of AR in Wuhan exhibited significant seasonal variation, with the first peak was from March to May and the second peak occurring between September and October. Correlation analysis revealed a moderate positive correlation between the monthly average SV of “Allergic Rhinitis” and “Mites” and “Mites + Pollen Allergy” in Wuhan, a weak positive correlation between the monthly average SV of “Allergic Rhinitis” and “Pollen Allergy,” and a positive correlation between monthly SV of “Allergic Rhinitis” and the meteorological index of pollen allergy (MIPA).

**Conclusion:**

The attention given to the topic on the internet, as measured by the search volume, was reflective of the situation in Wuhan, China. It has the potential to predict the epidemiological characteristics of AR and help medical professionals more effectively plan seasonal AR health education.

## Introduction

Allergic rhinitis is one of the most common allergic diseases in China (1). AR is a non-infectious chronic inflammatory disease of the nasal mucosa caused by an immunoglobulin E (IgE) mediated inflammatory response to inhaled allergens, often presented with sneezing, itching, rhinorrhea and nasal congestion (2). It has been proven that AR also affects people's sleep quality, social activities, learning and work efficiency, leading to decreased productivity (3) and life quality (4). According to recent epidemiological studies, AR has affected nearly 200 million people in China (5). Moreover, the prevalence of AR has been still on the rise in recent decades, and AR has become a serious public health, medical and economic problem.

Avoiding exposure to inhaled allergens can relieve the symptoms and reduce the incidence. Wearing face masks is an effective way, which most people have been doing since the COVID-19 pandemic. Recent research has shown that COVID-19 lockdowns have been proposed as contributing to decreasing symptom severity in patients with AR (6). At present, the global pandemic of COVID-19 has infected more than 230 million people (7). To prevent infection, staying at home and wearing a face mask are the most direct and effective ways. During the pandemic, people were highly mobilized and followed the commands of the government (8–11). Most people avoided crowded places and wore face masks when outside homes. Especially in Wuhan, where the COVID-19 pandemic first broke out and China imposed its first lockdown, people kept staying at home until the lockdown was lifted. And until now, most people in Wuhan consciously wear face masks in public.

With the popularization of Internet devices and health education, mobile phones and computers have become a very convenient way to obtain medical information and consult on health problems (12). Many studies (13–15) have shown that the frequency of disease queries and related symptom keywords is strongly correlated with the severity of the symptoms and could reflect the true trend of searchers' needs. For example, big data based on the Google platform can reflect and predict the prevalence trend of AR in the United States (13). In China, 92.1% of the SV data is on Baidu's search platform, and the usage of Baidu's platform accounts for 93.1% of the search service usage (16). Baidu Index is a big data analysis platform provided by Baidu based on the data generated by a large number of users' search behaviors. It takes keywords as the statistical object and calculates the search volume of each keyword, which is finally shown to the users through a variety of intuitive and clear curve graphics. The Baidu Index has been proven to be feasible in monitoring and predicting the epidemiological characteristics of diseases (17), and can reflect the real needs of searchers to a certain extent (15, 17, 18). The Internet search data is able to provide guidance to medical and health professionals, contributing to making targeted disease prevention and control as well as health education (15).

Pollen and dust mites are the most common allergens associated with AR in Wuhan (19, 20). However, it is difficult to predict dust mite levels, and there is no pollen concentration prediction in Wuhan yet. Therefore, we replace pollen concentration with MIPA, which can reflect the real-time sensitization effect of pollen concentration on human citizens. In addition, for research purposes, we gathered search data on dust mite-related terms. According to the China Meteorological Administration, MIPA is defined as the level of meteorological conditions' influence on pollen allergy. Based on the observed pollen concentration in the Hubei area, we graded the pollen concentration grade as the basic levels of MIPA in Wuhan. Then, the levels of MIPA after modification of meteorological conditions are used as the final MIPA.

In this study, we collected “Allergic Rhinitis” and various allergen search data in Wuhan as online public attention toward allergic rhinitis. This study aimed to analyze the AR-related search trends and online public attention in the Wuhan area, as well as the people's demand. We also gained meteorological data in Wuhan to analyze the relationship between online attention toward AR and the MIPA, partly verifying the importance and feasibility of developing pollen concentration prediction. Given the inadequacy of traditional methods and the lack of data sources, Internet search data is able to provide new insights into the epidemiological characteristics of AR, improve the detection and prediction of AR, and track public interest in multiple health topics. Thus, medical practitioners can fully utilize Internet data to track AR prevalence and patients' needs to create related health care policies and health education.

## Materials and methods

### Keywords selection and data retrieval

This study mainly analyzed the temporal search trends of AR and AR-related terms in Wuhan. To reduce the results bias caused by different language habits, we selected the most frequently used keywords related to AR on the web, including “Allergic Rhinitis” and AR-related search terms, “Pollen Allergy,” “Dust Mites,” “Mites,” “Dust Mites Allergy,” “Mites Allergy,” and “Mites Allergy + Pollen Allergy.” And the daily search volume of the above terms was counted from 1 Jan 2014 to 31 Dec 2021 through the Baidu Index platform (15, 18, 21). Because the common allergens causing AR in China are dust mites and airborne pollen, we select the keywords “Mites allergy + Pollen Allergy” as the main allergens of concern to AR patients.

We obtained the monthly basic level of MIPA in Wuhan from the Meteorological Industry Standard of the People's Republic of China–QX/T324-2016 Meteorological Index of Pollen Allergy (22) issued by the China Meteorological Administration in 2016. The fixed-point meteorological data, such as daily temperature, relative humidity, wind speed and precipitation, was provided by the Wuhan Meteorological Bureau, which contributed to revising the basic levels of MIPA by adding or subtracting levels.

Besides, we also collected user demand graph data from the Baidu Index platform, which can partly and intuitively reflect what AR patients were usually concerned about.

### Statistical analysis

The main variables examined were the search volume of AR, AR-related terms in Chinese and MIPA from 1 Jan 2014 to 31 Dec 2021.

Normality Test To verify the distributive normality of a data set, we used the Shapiro-Wilk test plus a graphical check of histograms and quantile-quantile diagrams.

Pearson and Spearman Cross-Correlations, when the data sets were normally distributed, the Pearson correlation R was used; otherwise, the Spearman correlation R was used. The correlation strength was assessed independently of the *P* values.

*P* values were used as a continuous measure of the strength of evidence against the null hypothesis. There were three independent hypotheses tested on a set of data in our study, namely the correlation between the monthly average SV of “Allergic Rhinitis” and “Mites,” “Allergic Rhinitis” and “Pollen Allergy,” “Allergic Rhinitis” and “Mites + Pollen Allergy” in Wuhan from 2014 to 2021, with *P* values corrected according to Bonferroni correction.

Software SPSS 26.0 (IBM Corporation, Armonk, NY, United States) was used for data analysis.

## Results

### Epidemiological characteristics of AR in Wuhan

**Figure 1:**
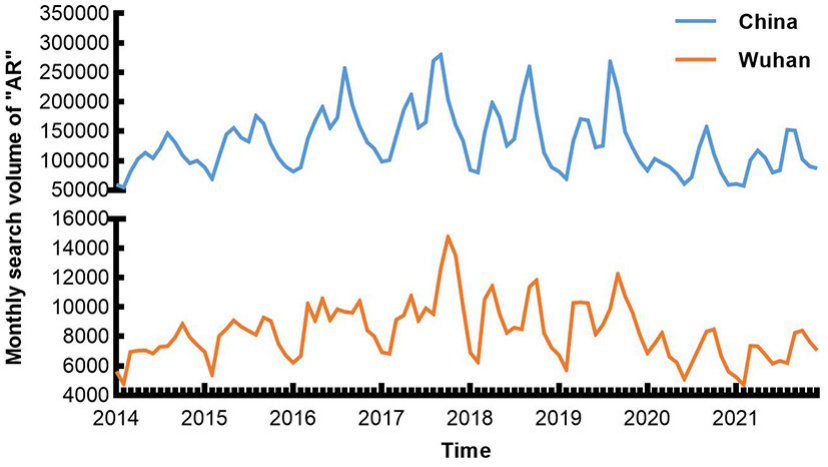
The yearly search volume of “AR” in China and Wuhan through Baidu search engine from 2014 to 2021.

**Figure 2:**
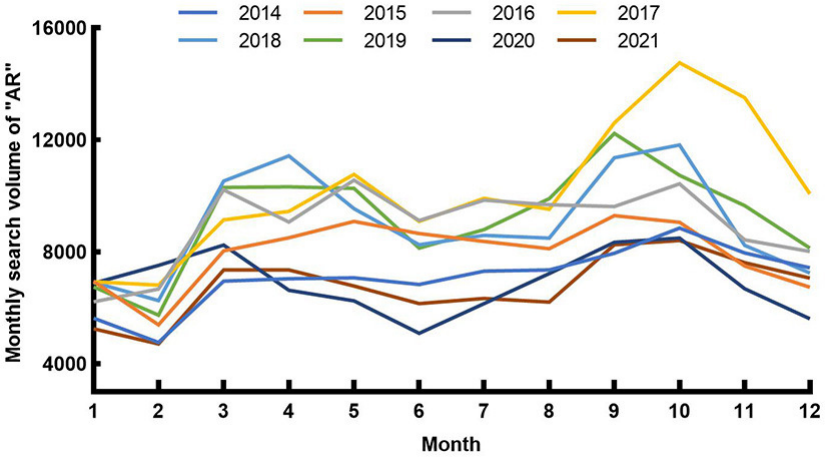
Epidemiological characteristics of “AR” in Wuhan from 2014 to 2021.

From 2014 to 2017, the Internet attention to AR in China and Wuhan both continued to rise (Figure 1). The yearly average Baidu search volume of “Allergic Rhinitis” in China was 101,668, 124,868, 154,793, and 175,793, respectively and 7,091, 7,970, 8,983, 10,209, respectively in Wuhan. And it remained at a high level from 2018 to 2019. Since 2019, AR Internet attention in China and Wuhan has shown a declining trend. Meanwhile, the valley values and the peak values also gradually decreased after the COVID-19 pandemic (Figure 1). Compared with the same period from 2015 to 2019, the SV of “Allergic Rhinitis” in Wuhan decreased significantly in 2020 and 2021 (Figure 2).

With monthly SV as the ordinate and the month as the abscissa, the Baidu users' Internet attention to AR in Wuhan reached its obvious peaks from Sep to Oct every year and small peaks from Mar to May every year (Figure 2). Meanwhile, the periods when these two seasonal peaks appeared were the same as in the whole country, and they also fell in line with airborne pollen in Wuhan (23).

### Internet attention correlation analysis of “Allergic Rhinitis” and AR-related keywords in Wuhan

**Figure 3:**
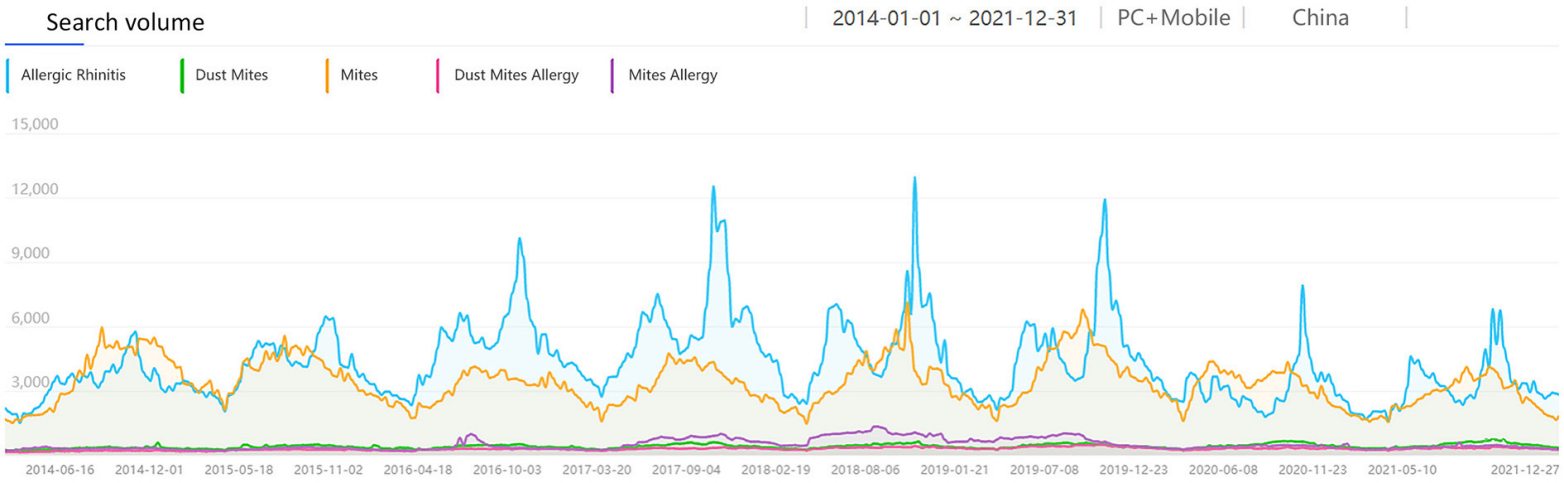
The search volume of “Mites” related keywords and “AR” in China from 2014 to 2021.

**Figure 4:**
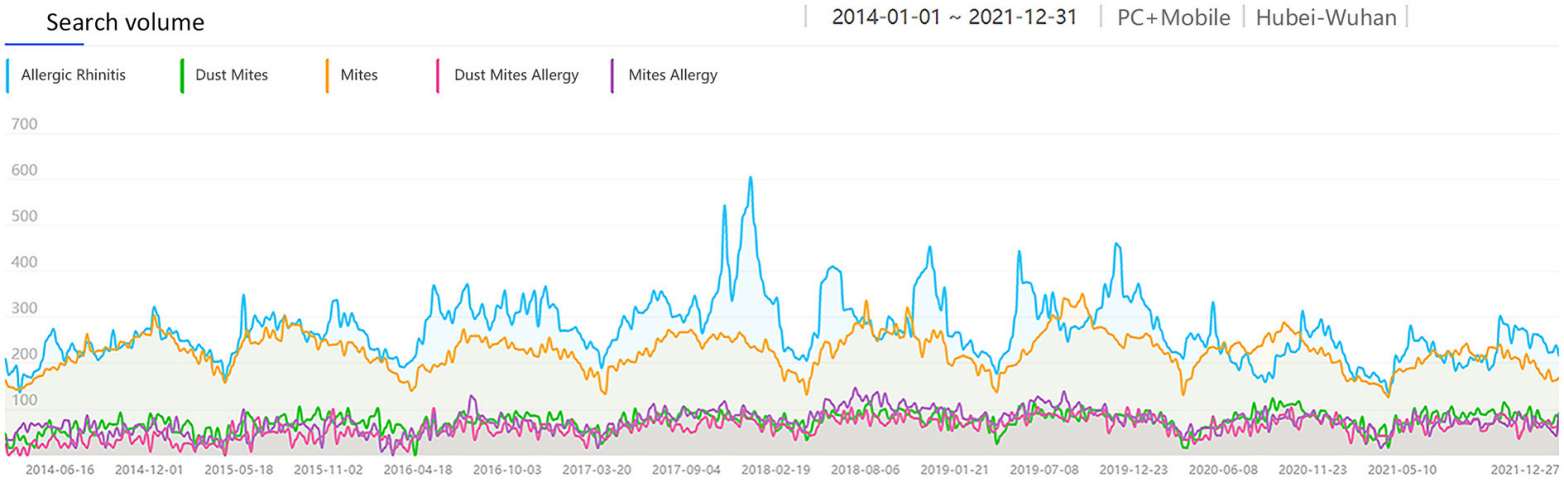
The search volume of “Mites” related keywords and “AR” in Wuhan from 2014 to 2021.

**Figure 5:**
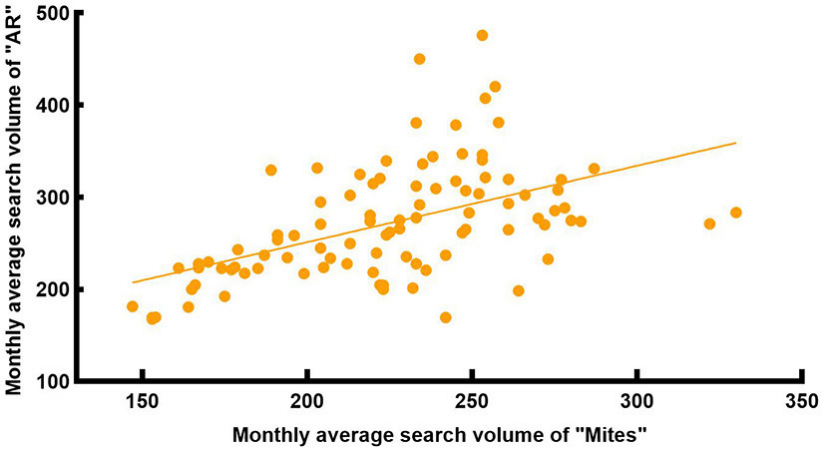
Correlation between the search volume of “AR” and “Mites” in Wuhan from 2014 to 2021.

Considering people's awareness of dust mite allergens and different language habits, we collected the search volume of AR-related keywords that people might search for. It included SV of “Mites,” “Mites Allergy,” “Dust Mites,” and “Dust Mites Allergy” from 1 Jan 2014 to 31 Dec 2021 in Wuhan and China. Among them, the SV of “Mites” was the highest in both national and Wuhan data, while the SV of other keywords was very low and had no obvious correlation with AR SV (Figures 3, 4). So, we collected the monthly average SV in Wuhan of “Allergic Rhinitis” and “Mites” from 1 Jan 2014 to 31 Dec 2021 for correlation analysis, and found a moderate positive correlation [R = 0.578, corrected *P* < 0.001, 95% CI: [0.419, 0.718], Figure 5] between the monthly average SV of “Allergic Rhinitis” and “Mites.”

**Figure 6:**
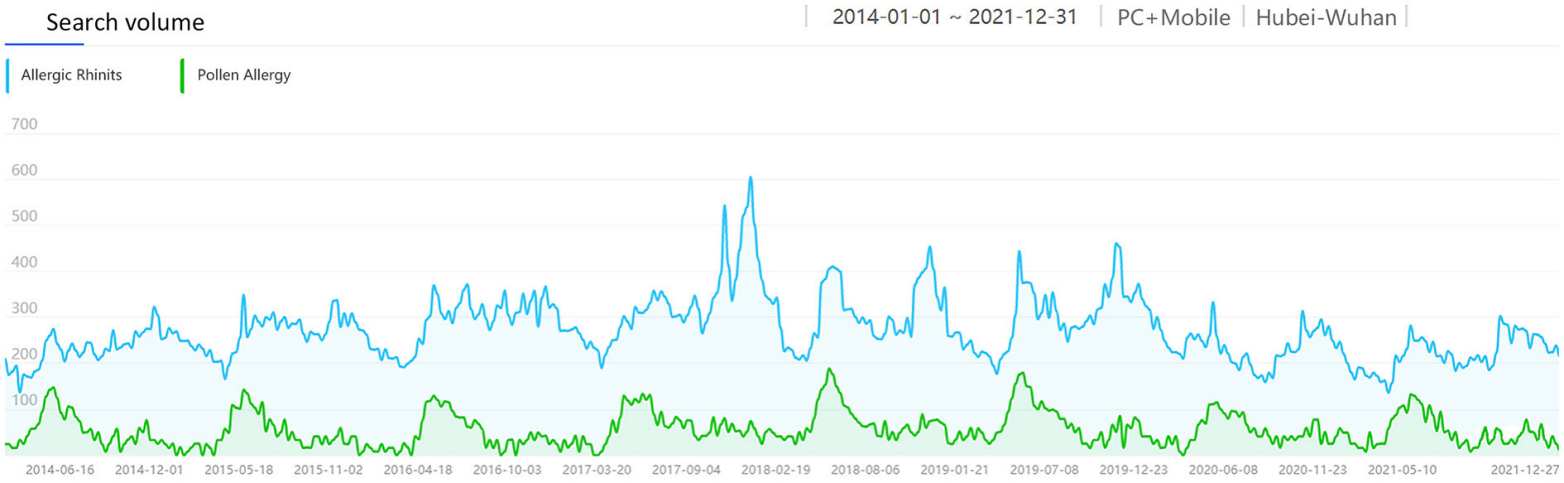
The search volume of “Pollen Allergy” in Wuhan from 2014 to 2021.

**Figure 7:**
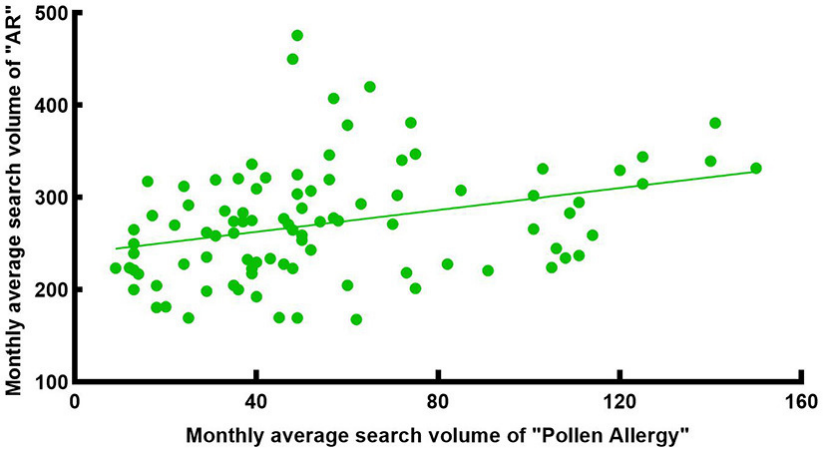
Correlation between the search volume of “AR” and “Pollen Allergy” in Wuhan from 2014 to 2021.

**Figure 8:**
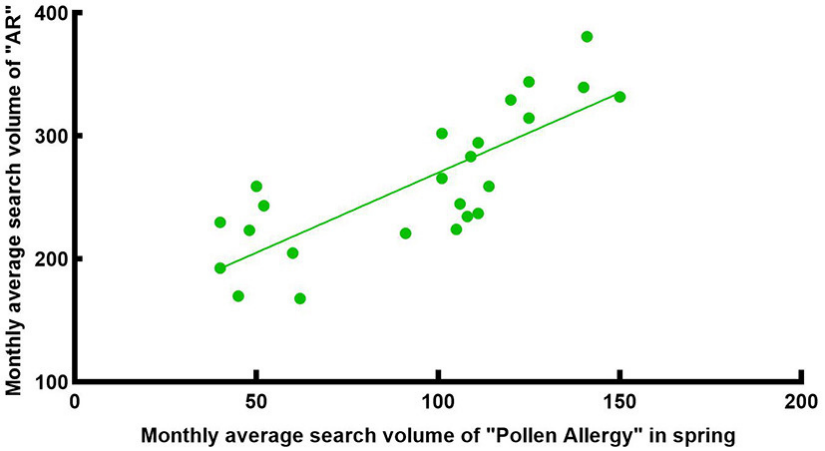
Correlation between the search volume of “AR” and “Pollen Allergy” in spring from 2014 to 2021.

**Figure 9:**
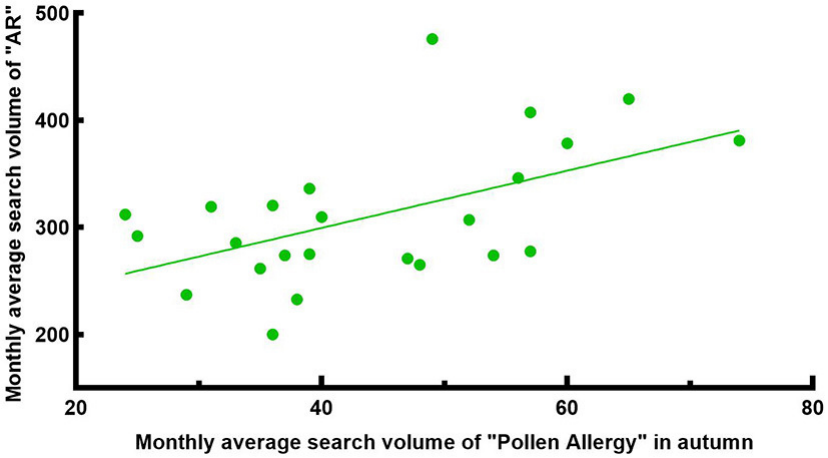
Correlation between the search volume of “AR” and “Pollen Allergy” in autumn from 2014 to 2021.

**Figure 10:**
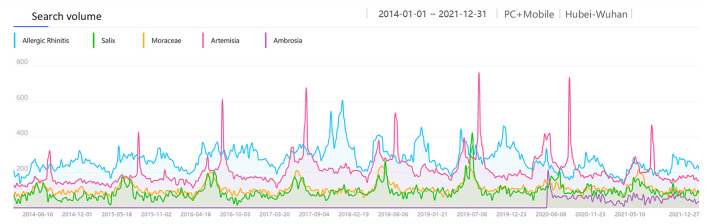
The search volume of various airborne pollens in Wuhan from 2014 to 2021.

Pollen is one of the most common allergens. Research shows (23) that airborne pollen in Wuhan had two peak periods throughout the year. The first peak was from Mar to Apr, and the second peak was from Aug to Oct. The airborne pollen in spring was mainly from Moraceae, Salix, Pendula, Cupressaceae, Pinus, while in autumn it was from Artemisia, Humulus and Ambrosia. We intercepted the monthly average search volume of the keywords “Allergic Rhinitis” and “Pollen Allergy” from 1 Jan 2014 to 31 Dec 2021 in Wuhan (Figure 6) for correlation analysis, which showed a weak positive correlation [R = 0.378, corrected *P* < 0.001, 95% CI: [0.187, 0.587], Figure 7]. There was a significant peak in the SV of “Pollen Allergy” in spring, while the SV in autumn was not high. Therefore, we analyzed the correlation between the SV of “Allergic Rhinitis” and “Pollen Allergy” in spring (Feb-Apr) and autumn (Aug-Oct), respectively. The results showed that the SV of the two keywords in Wuhan was strongly correlated in spring [R = 0.806, *P* < 0.001, 95% CI: [0.558, 0.911], Figure 8] and moderately correlated in autumn [R = 0.541, P = 0.006, 95% CI: [0.252, 0.750], Figure 9]. Otherwise, the SV trends of various airborne pollens in Wuhan (Figure 10) were almost consistent with the pollen season and showed that people knew little about certain airborne pollens (e.g., ambrosia, etc.).

**Figure 11:**
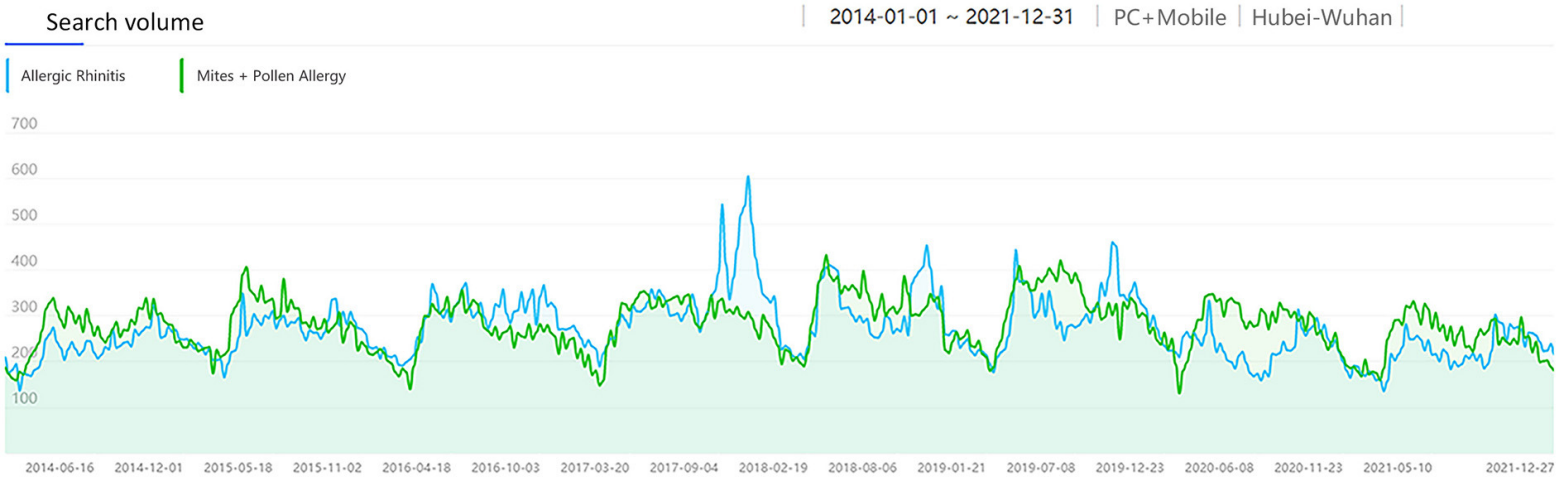
The search volume of “AR” and “Mites + Pollen Allergy” in Wuhan from 2014 to 2021.

**Figure 12:**
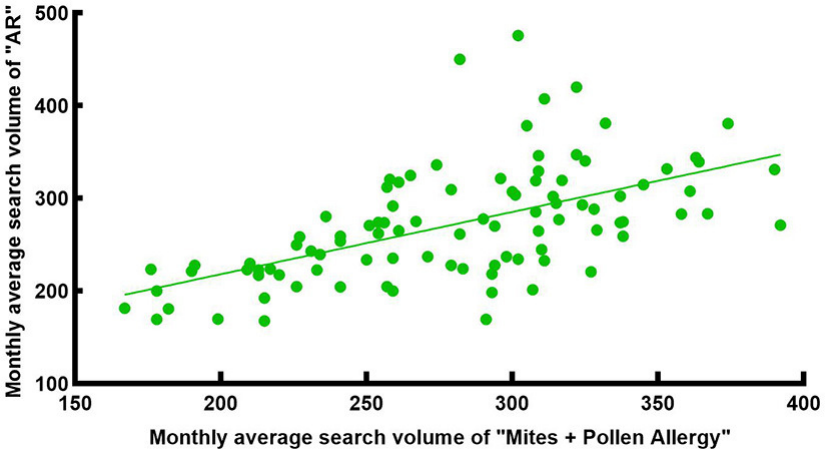
Search volume correlation analysis of “AR” and “Mites + Pollen Allergy” in Wuhan from 2014 to 2021.

As a city located in central China, the most common allergens causing AR in Wuhan include both pollen and dust mites. Therefore, we analyzed the correlation between the monthly average SV in Wuhan of “Allergic Rhinitis” and the SV of “Mites + Pollen Allergy” (Figure 11), representing the combined SV of the two keywords, and got a moderate positive correlation [R = 0.635, corrected *P* < 0.001, 95% CI: [0.501, 0.738], Figure 12].

### Internet attention correlation analysis of “Allergic Rhinitis” and the meteorological index of pollen allergy in Wuhan

The correlation between monthly Internet attention to “Allergic Rhinitis” and the MIPA in Wuhan from 1 Jan 2014 to 31 Dec 2021 was shown in Table 1. Monthly SV showed a positive correlation with MIPA in 2014 [R = 0.370, *P* = 0.236, 95% CI: [−0.215, 0.900]], 2015 [R = 0.708, *P* = 0.010, 95% CI: [0.145, 0.950]], 2016 [R = 0.538, *P* = 0.071, 95% CI: [−0.126, 0.927]], 2017 [R = 0.378, *P* = 0.226, 95% CI: [−0.170, 0.884]], 2018 [R = 0.900, *P* < 0.001, 95% CI: [0.641, 0.975]], 2019 [0.911, *P* < 0.001, 95% CI: [0.684, 0.965]], 2020 (0.780, *P* = 0.003, 95% CI: [0.283, 0.962]], 2021 [0.731, *P* = 0.007, 95% CI: [0.296, 0.901]], and 2014–2021 [0.554, *P* < 0.001, 95%CI: [0.391, 0.686]].

**Table 1:** Correlation between the monthly search volume of “Allergic Rhinitis” and “the meteorological index of pollen allergy” in Wuhan from 2014 to 2021.

**Year**	**Monthly search volume ($\bar{\text{X}}\pm$s)**	**MIPA (M ±IQR)**	**R value**	***P* value**
**2014**	7,091.00 ± 1,065.34	1.50 ± 2.60	0.370	= 0.236
**2015**	7,970.42 ± 1,149.26	2.00 ± 2.00	0.708	= 0.010
**2016**	8,983.33 ± 1,413.96	2.00 ± 2.76	0.538	= 0.071
**2017**	10,208.5 ± 2,401.61	2.00 ± 2.75	0.378	= 0.226
**2018**	9,051.5 ± 1,872.10	2.00 ± 2.65	0.900	< 0.001
**2019**	9,240.67 ± 1,815.21	2.00 ± 2.75	0.911	< 0.001
**2020**	6,921.33 ± 1,084.13	2.00 ± 2.00	0.780	= 0.003
**2021**	6,787.00 ± 1,119.48	2.50 ± 2.75	0.731	= 0.007
**2014–2021**	8,281.72 ± 1,918.77	2.00 ± 2.00	0.554	< 0.001

### Demand graph of AR

**Figure 13:**
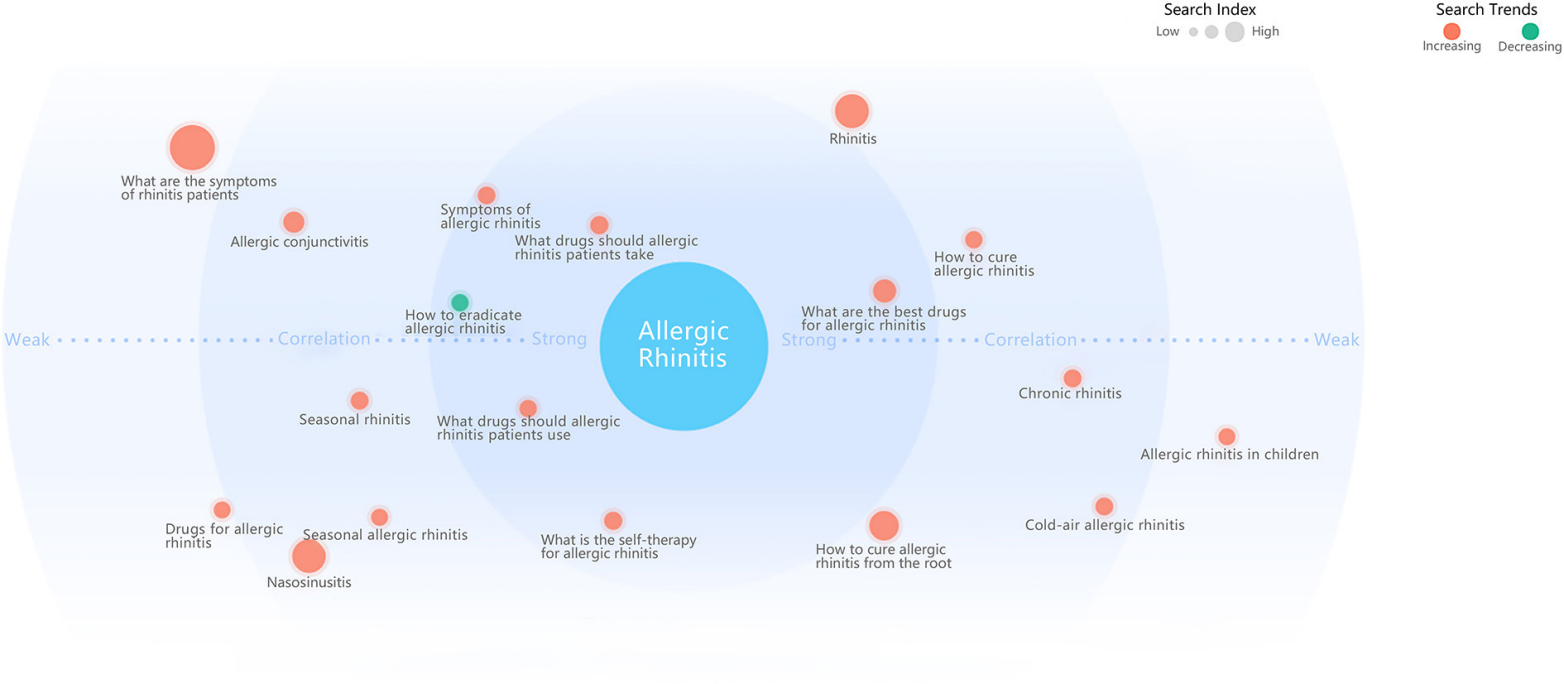
Demand graph of AR.

The demands for relevant search terms were reflected in the changes of users' search behavior. The comprehensive calculation of keywords and the correlation degree of related words, as well as the degree of the search demands of related words, were visualized on the Demand Graph (Figure 13). The Demand Graph showed that searchers were most concerned about “How to cure allergic rhinitis from the root,” “Rhinitis,” and “What are the symptoms of AR patients.” And the search terms that were most relevant to “Allergic Rhinitis” included “What drugs should AR patients take,” “What is the self-therapy for AR” and “Symptoms of allergic rhinitis.” Besides, there were some moderately related words to “Allergic Rhinitis,” including “Allergic conjunctivitis,” “Seasonal allergic rhinitis,” “Chronic rhinitis,” “Allergic rhinitis” in children” and “What to do when allergic rhinitis patients have eye itching” and so on.

## Discussion

### Principal results

With the rapid development of the Internet and modern information technologies, it is quite common to use Internet data to monitor people's anxiety, happiness, mood, and even the risk of suicide in a certain area (24–26). In the medical field, Google Trends, used for surveillance of disease outbreaks in 2009, has become a model of big data application (27), which uses aggregated Google search data to estimate the current global influenza epidemic in almost real time (28). As the Internet could supply the simplest and primary source of health care information, patients are more likely to have a preliminary consultation online before seeing a doctor (28). To date, Chinese research of search engine data is mainly based on the Baidu Index. There have been some Baidu Index-based relevant studies on the prediction of the brucella epidemic, kidney stones, AIDS, hand-foot-mouth disease, and other diseases (14, 15, 17, 29), which all showed quite good accuracy. Therefore, studying the infodemiology and infoveillance characteristics of AR might help to understand the actual epidemic trend and attention to patients to a certain extent (14).

At present, there has been a little AR-relevant research based on the Baidu Index in China, such as research on the temporal and spatial characteristics of AR epidemics (30), the correlations between search volume and the outpatient visit volume of AR in Beijing and Guangzhou (31), and the epidemiological relevance between adenoid hypertrophy and AR (32), and all the research partly verifies the feasibility of analyzing AR features from the Baidu Index. However, there is no web-based research on the epidemiological characteristics of AR in Wuhan or its association with the COVID-19 pandemic, nor on the people's awareness of allergic rhinitis or demand for AR through demand graph analysis. Meanwhile, as a city located in central China, Wuhan has seen rapid economic development in recent years, but there is a lack of current data on allergic diseases. During the COVID-19 pandemic, almost everyone in Wuhan was quarantined at home for at least 3 months, which made it very inconvenient to go to see a doctor and helped people develop the good habit of wearing face masks outdoors. In recent years, mobile phones and computers have become the preferred methods of obtaining medical information. Therefore, the Baidu Index platform may provide information about the incidence of AR in Wuhan and contribute to understanding the needs of patients.

In this study, we found that the Internet attention to AR in Wuhan and China gradually increased from 2014 to 2017 and remained at a high level from 2018 to 2019. After Wuhan was under lockdown on 23 Jan 2020 due to the COVID-19 pandemic, the Internet attention to AR in Wuhan gradually decreased. These trends are consistent with the reported development trends of AR incidence (4, 33), which indicates that the Internet attention to AR based on search data can reflect the prevalence trend of AR to some extent. Meanwhile, the data released by Nanshan Zhong, the leader of the National Health Commission Senior Expert Group in China, showed that the incidence of all 40 legal infectious diseases in China has decreased since the first half of 2020. Hence, it can be conclude that good hygiene habits, such as maintaining social distance and wearing face masks outdoors regularly, play an integral role in preventing COVID-19 pandemics and associated infectious diseases, as well as reducing the incidence and attacks of AR, which has been confirmed by studies (33–35). Thus, we suggest that AR patients should better maintain good personal hygiene habits and wear face masks outdoors, which can effectively protect them from AR attacks.

We also found that the Baidu search volume of AR in Wuhan showed significant seasonal variation. The first peak was from Mar to May and the second peak was from Sep to Oct, which was in line with the reported two peaks of airborne pollen in Wuhan (23). The two peaks are seasons for airborne pollen in Wuhan, when clinical symptoms are most pronounced and severe in AR patients, which suggests that pollen is an important allergen for people in Wuhan, though most studies based on allergen detection experiments report that mites are the most common allergen. Therefore, we can publish more targeted information about AR prevention and treatment on the Internet during the relevant time period. This, in turn, can enhance patient compliance with treatment and demonstrate the importance of health education in the AR prevention and treatment system.

In addition, there was a moderate positive correlation between the SV of “Allergic Rhinitis” and “Mites” in Wuhan, indicating that the AR patients in Wuhan are comparatively concerned about mites as a kind of allergen, and a considerable number of them searched for “Dust Mites” and “Allergic Rhinitis” at the same time to acquire knowledge of allergen prevention and control. Besides, the SV of “Allergic Rhinitis” and “Pollen Allergy” in Wuhan was strongly correlated in spring (Feb-Apr) and moderately correlated in autumn (Aug-Oct). The SV of the main spring airborne pollens in Wuhan, Moraceae and Salix, had obvious peaks in spring (Figure 10). Interestingly, the SV of the main autumn airborne pollen, Artemisia, had two peaks in spring too, which was due to the Chinese custom of using Artemisia during the Dragon Boat Festival in early June every year. As shown in Figure 10, people were also unaware of certain airborne pollens (e.g., ambrosia, etc.). Consequently, people in Wuhan need to raise their awareness of airborne pollen that can cause AR. A moderate positive correlation between the SV of “Allergic Rhinitis” and “Mites + Pollen Allergy” indicates that people in Wuhan are fully aware of AR and pay high attention to the related allergens.

To sum up, our study showed the trend characteristics of “Allergic Rhinitis” search volume are basically consistent with the results of previous epidemiological investigations (4, 5, 23, 33–35) and the correlations of “Allergic Rhinitis” and “Pollen Allergy,” “Dust Mites Allergy,” and “Mites” SV are the same as the assumption. It suggests that Baidu Index data has the potential to reflect and predict the prevalence of AR by analyzing users' search behavior and medical information needs, and partly guide AR health education in Wuhan, which is consistent with previous research that found Google trends can reflect the epidemiological characteristics of AR in the United States (13).

Pollen-induced AR has attracted more and more attention to people in China. This study found a positive correlation between the monthly SV in recent years and the MIPA. Pollen concentration plays a key role in the outbreak of AR and predicting pollen concentration in advance is very important to control AR. However, there are very few cities in China to carry out pollen concentration predictions (Beijing, Tianjin). Therefore, we select the MIPA instead of pollen concentration in Wuhan, which can reflect the real sensitization effect of pollen concentration on the human body. The positive correlation of AR Internet attention and the MIPA suggests that we can use the Baidu Index to develop a calculation model to predict the arrival and duration of pollen season, which is a real-time and simple method of self-health management for patients with AR. When the SV of “Allergic Rhinitis” begins to increase, which indicates the arrival of pollen season, and pollen-sensitized AR patients should be reminded to take active measures to prevent AR, such as wearing face masks or using medication. This will not only contribute to avoiding the onset of seasonal AR, reducing the pain of patients, but also help to save the expenditure of social medical resources. Of course, more studies are needed to confirm the relationship between SV of “Allergic Rhinitis,” MIPA in different cities and actual pollen concentration.

Besides, we also collected user demand graph data, which can partly and intuitively reflect what AR patients were usually concerned about. As shown in the Demand Graph of allergic rhinitis, patients are most concerned about the therapy and symptoms of AR, and most AR patients search for “Nasosinusitis,” “Chronic rhinitis,” “Allergic conjunctivitis,” and “Eye itching.” Besides, the SV of related words, such as “Seasonal allergic rhinitis,” “Allergic rhinitis in children,” and “Can allergic rhinitis infect,” is increasing. As can be seen from the Demand Graph, many AR patients are still not cured or relieved and are looking for a radical cure. In addition, a considerable number of AR patients are suffering from nasosinusitis and allergic conjunctivitis at the same time. In general, people would like to search for medical information about AR more and more.

Our results are based on Internet search data, which reflects the public awareness of AR in a comparatively objective way. However, research has shown that mass media have an impact on the search behavior of web users and play an important role in managing an infodemiology and conditioning the search behavior of web users (36, 37). Many users first acquire some knowledge of AR through media and form an initial understanding, such as learning the main symptoms of AR from videos and articles shared on social media platforms, common allergens causing AR in life and treatments for AR. Some people resonating with the knowledge of AR wonder whether they have AR or become interested in AR. These web users then search for AR-related terms through all kinds of search platforms to gain insight into the disease. Indeed, the words used in media coverage will affect the search terms chosen by web users (38), and people may search more due to more media coverage during the high season of AR. Besides, the majority of media popularization of science is conducted by medical professionals, providing specialized terminology and knowledge related to diseases, but there is still a small amount of misinformation without scientific basis, leading to bias in some search terms (39, 40). This bias has been taken into account and we have eliminated the effect of it on the results by searching multiple synonyms. In fact, due to the lack of public education about AR through the media in most cities in China, the impact of the mass media on the search behavior of web users is minimal. Nevertheless, these influences are mostly beneficial for the population, which inspires us to better guide our target audience with the help of web-based social media.

### Limitations

The main limitations of this study should be addressed. First, Baidu Index only analyzes search data from Baidu, not social media platforms or other search engines, and provides limited data. Moreover, the impact of mass media coverage on web users' behavior is difficult to assess and eliminate. And these search behaviors may be also influenced by the diagnostic ability of the consulted doctors, such as the general practitioners' ability to distinguish between allergic rhinitis and non-allergic rhinitis. Second, the primary users of search engines are young and middle-aged people, which may lead to an age bias. The Internet and smartphone penetration rates in economically developed areas are higher than those in non-developed areas, resulting in a certain degree of regional bias. But with the development of the economy and the popularization of the Internet, the age-bias and regional bias will gradually decrease. Third, whether predicting the arrival of pollen season or the attacks of AR based on Internet search data, there must be a lag. We still need to study the approximate lag time further, and then bring the results forward when making predictions. Furthermore, information about AR patients could only be based on age, gender, and regions, but information such as the users' age and gender in a certain region, socioeconomic status, ethnicity, and educational background could not be obtained. More studies are needed in the future to adequately demonstrate the role of big data in understanding the population's needs and in the surveillance of diseases.

### Comparison with prior work

To the best of our knowledge, this is the first study that analyzes the epidemiological characteristics of AR in Wuhan as well as the correlation between MIPA and the prevalence of AR based on the Baidu Index. This is also the first web-based research on the association of the Internet search trends with the COVID-19 pandemic. In recent years, the number of active users of Baidu has been growing, so Baidu's big data has great potential in medical treatment and disease epidemic surveillance and will play a more and more important role.

## Conclusion

In summary, our study showed that Baidu Index data could reflect the real-world situation to some extent and has the potential to predict the epidemiological trends of AR. The Internet data can also be used by medical practitioners to monitor the prevalence of AR and the patients' needs, provide guidance to make disease-specific health care policies and health education, and optimize clinical consultations. Furthermore, developing a platform to predict pollen concentrations as well as the arrival and duration of the pollen season is an excellent method of prevention and management for AR patients. More importantly, keeping good personal hygiene habits and wearing face masks outdoors can help reduce the incidence and attacks of AR.

## Data availability statement

The datasets presented in this study can be found in online repositories. The names of the repository/repositories and accession number (s) can be found in the article/supplementary material.

## Author contributions

YW and ZG collected and analyzed the data, discussed the detail, and wrote this article together. HL performed the statistical analysis. YX supervised the study, modified this manuscript, and was major contributor in writing the manuscript. All authors read and approved the final manuscript.

## Funding

This research was supported by the National Natural Science Foundation of China (No. 82071017), the Natural Science Foundation of Hubei Province (No. 2021CFB125), and the Fundamental Research Funds for the Central Universities (No. 2042021kf0093).

## Conflict of interest

The authors declare that the research was conducted in the absence of any commercial or financial relationships that could be construed as a potential conflict of interest.

## Publisher's note

All claims expressed in this article are solely those of the authors and do not necessarily represent those of their affiliated organizations, or those of the publisher, the editors and the reviewers. Any product that may be evaluated in this article, or claim that may be made by its manufacturer, is not guaranteed or endorsed by the publisher.
